# A 12-Month Digital Peer-Supported App Intervention to Promote Physical Activity Among Community-Dwelling Older Adults: Follow-Up Study of a Nonrandomized Controlled Trial

**DOI:** 10.2196/66610

**Published:** 2025-05-28

**Authors:** Kento Tabira, Yuko Oguma, Shota Yoshihara, Megumi Shibuya, Manabu Nakamura, Natsue Doihara, Akihiro Hirata, Tomoki Manabe, Takashi Yamashita

**Affiliations:** 1 Sports Medicine Research Center, Keio University, 4-1-1 Hiyoshi, Kohoku-ku, Yokohama, Kanagawa, 223-0061, Japan, 81 45-566-1090; 2 Graduate School of Health Management, Keio University, Fujisawa, Kanagawa, Japan; 3 Department of Rehabilitation Sciences, Graduate School of Medical Sciences, Kitasato University, Sagamihara, Kanagawa, Japan; 4 A10 Lab Inc, Chuo-ku, Tokyo, Japan; 5 Japan Society for the Promotion of Science, Chiyoda-ku, Tokyo, Japan; 6 Department of Sociology, Anthropology, and Public Health, Gerontology Doctoral Program, University of Maryland Baltimore County, Baltimore, MD, United States

**Keywords:** physical activity, exercise, exercising, physical function, older adult, aging, eHealth, peer support, mobile phone, mHealth, mobile health, mobile app, app, application, smartphone, digital

## Abstract

**Background:**

Mobile apps and peer support are known to effectively promote physical activity in older adults, which, in turn, improves physical function. Previously, we investigated the feasibility and impact of using digital peer-supported apps (DPSAs) to increase physical activity among older adults over a 3-month period. However, the long-term feasibility and impact on sustainable behavior change remain unknown.

**Objective:**

This study aims to evaluate the 12-month feasibility of the DPSA and to obtain preliminary estimates of its effects on physical activity and physical function among older Japanese adults.

**Methods:**

This nonrandomized controlled trial recruited older adults aged 65 years or older from 2 physical activity programs. Participants chose either the intervention (app program + exercise instruction) group or the control (exercise instruction only) group. Only those participants who had completed the 3-month intervention and wished to continue in the 12-month follow-up intervention study were included. DPSA feasibility was assessed using retention and adherence rates. Physical activity was assessed using accelerometers, capturing daily step count, light-intensity activity, moderate to vigorous intensity activity, and sedentary behavior. Physical function was evaluated using grip strength and the 30-second chair stand test (CS-30). Accelerometer measurements were collected every 3 months over 12 months (5 time points, including baseline), whereas physical function was measured at baseline, 3 months, and 12 months.

**Results:**

The follow-up study included 44 of 66 participants from the 3-month intervention study, with 26 participants in the intervention group and 18 participants in the control group. The 12-month retention rate for participants in the DPSA intervention group was 73% (19/26), whereas the retention rate among all 41 participants, including those who chose not to participate in the follow-up study, was 46% (19/41). The adherence rate was 85.9%. The average number of steps per day (95% CI) in the intervention group changed before and after DPSA use (*P*=.048). We observed an increase of 1736 (β=1736, 95% CI 232-3241) steps per day compared with baseline. No significant change was observed in the control group. There were significant within-group differences in CS-30 scores for both intervention (*P*<.001) and control (*P*=.03) groups over the 12-month period. Specifically, there was a significant change in CS-30 scores (95% CI) between the baseline and 12-month assessments for the intervention (β=6.5, 95% CI 3.8-9.1; *P*<.001) and control (β=3.8, 95% CI 0.6-7.1; *P*=.02) groups.

**Conclusions:**

Participants with long-term DPSA use observed increases in average daily steps and CS-30 scores before and after DPSA use, although only a limited number of older adults had long-term access to the DPSA. Identifying ways to expand long-term DPSA use among older adults is necessary. Additionally, randomized controlled trials should be conducted to determine the long-term effects of DPSAs on physical activity and function in older adults.

## Introduction

### Background

The world’s population is aging at an unprecedented rate [1]. The number of adults older than 65 years has tripled over the past 50 years, and by 2050, older adults are expected to account for a quarter of the global population [2-4]. Japan has a high proportion of older adults: 29.1% of the total population was aged 65 years or older in 2023 [5]. Healthy aging is a global health care challenge as population aging accelerates [6]. Regular physical activity aids in reducing the risk of noncommunicable diseases [7] and is associated with improved physical health [8] and increased life expectancy [9]. However, despite decades of public health interventions, the global physical activity level has remained stable or even declined, making it an important health policy challenge [10]. Given the world’s aging population and the health benefits of physical activity, it is critical to promote regular physical activity among older adults. The Japanese guidelines for physical activity [11] recommend a minimum of 15 metabolic equivalent (MET) hours per week of physical activity with an intensity of at least 3 METs in older adults. Physical activity of 15 MET hours per week can be converted into steps, which is more than 6000 steps per day [11]. However, few older adults meet this recommendation: among men, 45% of those aged 65‐74 years, 32% of those aged 75‐84 years, and 11% of those aged 85 years or older meet this recommendation, and among women, 38% of those aged 65‐74 years, 22% of those aged 75-84 years, and 5% of those aged 85 years or older meet this recommendation [11]. Regular physical activity improves physical function in older adults [12]. Declining physical function is linked with the loss of mobility and activities of daily life, which are core dimensions of physical disabilities [13,14], and thus, both physical activity and physical function need to be improved.

Recently, mobile apps have been successfully used to increase physical activity levels [15,16]. eHealth, or electronic health, encompasses health care services supported by information and communication technology, including computers, mobile phones, and satellite communications, for health services and information. Moreover, mHealth, or mobile health, refers to the use of smart or portable devices for providing health services and information [17]. These interventions for older adults have been shown to be effective in increasing the time spent in physical activity, energy expenditure in physical activity, and steps walked [18,19]. In a review comparing mHealth with face-to-face interventions, interventions that included mHealth were shown to have increased steps and total physical activity, but there was no observed difference in physical function [20].

In the systematic review by Duan et al [21], eHealth interventions for physical activity have shown that theory-based interventions are more effective than non–theory-based interventions. The transtheoretical model and social cognitive theory were the top 2 most frequently supporting theories, and the studies included in this systematic review with the largest effect sizes were based on these 2 theories [22]. The social cognitive theory proposed by Bandura [23] stipulates that behavior is learned by observing and imitating others. This process is called observational learning or modeling and has been extensively studied in the context of motor skill development and education [24-26]. Self-efficacy, an important aspect of social cognitive theory [23], is an crucial determinant of exercise persistence and outcomes; interventions based on self-efficacy can promote exercise participation [27].

The effectiveness of peer support interventions for physical activity is often explained by social cognitive theory [28]. Webel et al [29] defined peer support as “a method of teaching or facilitating health promotion that makes use of people sharing specific health messages with members of their own community." Our previous study using a digital peer-supported app (DPSA) framed by the social cognitive theory showed that the feasibility of the DPSA was adequate and that the number of daily steps and the level of moderate to vigorous intensity physical activity (MVPA) increased in older participants [30]. There are 2 main types of peer support [31]: the first includes methods related to education and information, such as peer tutoring and mentoring; the second is the emotional support provided by peers. Our research is based on peer support interventions that provide emotional support. Peer support is provided by comparable peers and promotes physical activity in ways that cannot be done by professionals or family members; Burton et al [32] reported that peer support increased adherence to an exercise program; Ginis et al [28] reported that peer support was as effective as professional intervention. Peer support may be cost-effective when considering the expense of paying professionals [33]. Peer support through the DPSA includes social support, which contributes to the success of eHealth and mHealth interventions for increasing physical activity among older adults [34]. In addition, DPSA interventions do not require in-person gatherings, thus reducing constraints owing to scheduling issues, meeting locations, and costs (eg, transportation) [35]. Thus, the DPSA may be effective in promoting physical activity among older adults. However, our previous study was a short-term intervention of 3 months, and the long-term feasibility and impact for sustainable behavior change remains unknown.

Three of 4 review studies concluded that mHealth or eHealth interventions are effective over short term (1‐6 months) in promoting physical activity in adults aged 50 years or older [34]. All 3 reviews incorporated randomized controlled trials (RCTs) comparing interventions that were not eHealth or mHealth (eg, paper-based intervention, professional face-to-face intervention, and group face-to-face intervention), or no intervention. Despite the demonstrated long-term health benefits of physical activity [36], long-term empirical evidence of mHealth and eHealth, beyond 6‐12 months, remains scarce [37-39]. Furthermore, no study has continued the app intervention for 12 months and collected device-based physical activity measures in community-dwelling older adults older than 65 years [40]. Physical activity interventions for older adults often face challenges regarding long-term participation owing to age-related health decline, low self-efficacy, and poor geographic access to physical activity spaces [41,42]. There is a need to test the long-term effectiveness of the DPSA in promoting physical activity among older adults. However, before testing the long-term effectiveness of the DPSA on a large scale, a reasonable first step is to examine the feasibility and preliminary changes in physical activity, physical function, and self-efficacy in community settings. We hypothesized that 1 year of DPSA use would increase physical activity owing to increased self-efficacy for exercise. We also expect that the increase in physical activity will be accompanied by an increase in physical function.

### Objectives

This study was a 12-month longitudinal study of participants in a 3-month DPSA intervention study who volunteered to participate in a follow-up study. The objectives of this study were twofold: (1) to evaluate the feasibility (retention and adherence rates) of using the DPSA to promote physical activity in older adults over a 12-month period, and (2) to measure preliminary estimates of the effects of physical activity, physical function, and self-efficacy for exercise through the use of the DPSA.

## Methods

### Study Design

This study is a nonrandomized controlled trial of 2 groups conducted over 12 months and is a follow-up study of a 3-month intervention trial [30]. This study was conducted in Fujisawa City, Kanagawa, Japan. Fujisawa City is in the southeastern part of Kanagawa and is an urban area close to Tokyo. As of April 2023, the city had a population of 445,291; of those, 24.5% (109,005) were aged 65 years or older. The percentage of older adults in the total population is increasing year by year [43]. This study was conducted as a collaboration between local governments, mobile app development companies, and universities. Industry-government-academia collaboration is important to further scientific research that is relevant to real-world community issues [44,45].

### Ethical Considerations

This study was approved by the research ethics committee of Sports Medicine Research Center at Keio University (approval no. 2022‐07). Informed consent for the follow-up study was obtained from all participants in the 3-month intervention study. The data obtained were anonymized. The study protocol was registered in the University Hospital Medical Information Network (UMIN000050618).

### Participants

The study included Fujisawa City residents aged 65 years or older. In Japan, older adults are generally defined as persons aged 65 years or older [40]. We recruited participants for two 3-month programs designed to increase physical activity [30]. Participants from 2 different areas within the municipality of Fujisawa City were recruited through flyers, local newsletters, and calls to related organizations. Participants chose either intervention (app program and exercise instruction) or control (exercise instruction only) group. The 3-month intervention study [30] included 74 participants (intervention group: 41, control group: 33). The follow-up study was introduced to 66 participants who completed the 3-month intervention. Participants (n=8) who did not provide their informed consent were excluded from the follow-up study. The eligibility criteria were adults aged 65 years or older who could walk independently and perform daily activities without being advised by a physician to refrain from physical activity (self-reported criterion). Prospective participants were screened using a personal health status questionnaire based on the Physical Activity Readiness Questionnaire [46-48] to ascertain any potential health problems with study participation. Because the purpose of this study was to assess feasibility and obtain preliminary estimates, sample size was not calculated; the number of participants was limited because the study was conducted in collaboration with the local government.

### Intervention

#### Program

**Figure 1.**
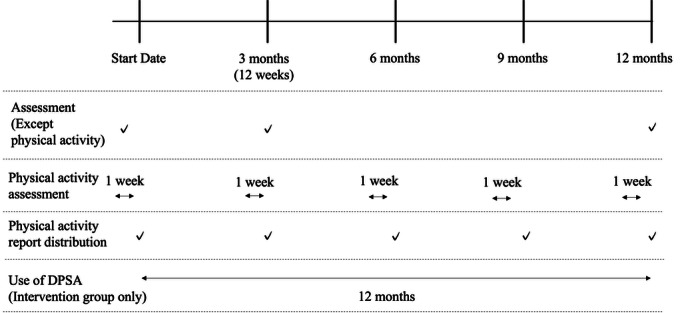
Timeline of 12-month intervention procedure. DPSA: digital peer-supported app.

Regardless of program selection, all participants underwent face-to-face exercise instruction, program introduction, and baseline assessment by a physical therapist or health fitness instructor. Exercise instructions focused on aerobic, stretching, muscle strengthening, and balance exercises based on the original “Fujisawa + 10 exercise” program [49,50]. The timeline of the study procedure is shown in Figure 1. Both intervention and control groups were instructed to increase their daily physical activities. The participants completed surveys and physical function measurements at baseline (start date), 3 months, and 12 months postintervention. Additionally, physical activity levels were measured every 3 months, 5 times in total, using triaxial accelerometers. Individualized physical activity reports were generated from the collected data and provided as feedback to the participants. The intervention group began using the app 1 week after the baseline outcome assessment.

#### Digital Peer-Supported App

**Figure 2.**
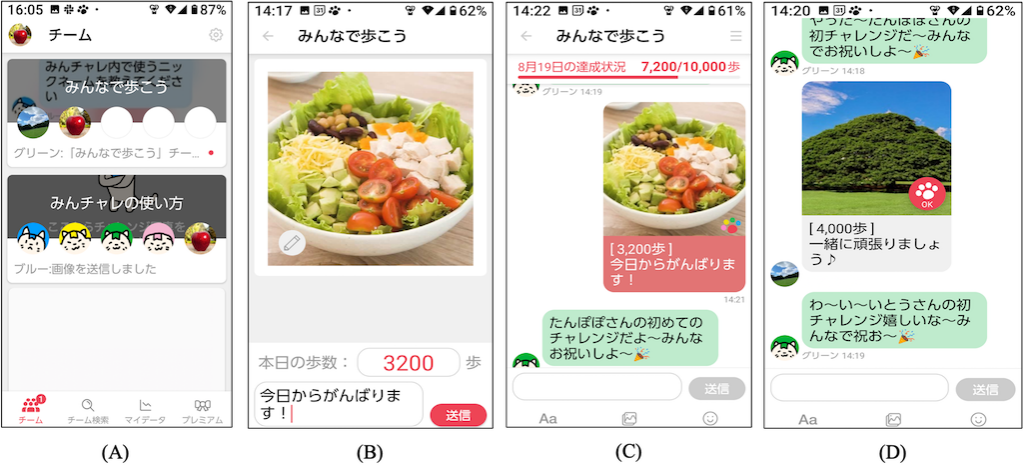
Examples of mobile app screens. (**A**) A group is selected. (**B**) Photographs, step count, and comments are posted on the group. A photograph taken that day is posted and comments are added on the day’s events. (**C**) Contents of the posts are displayed in the group. The total number of steps for the group is displayed. (**D**) Responses to posts by group members.

**Figure 3.**
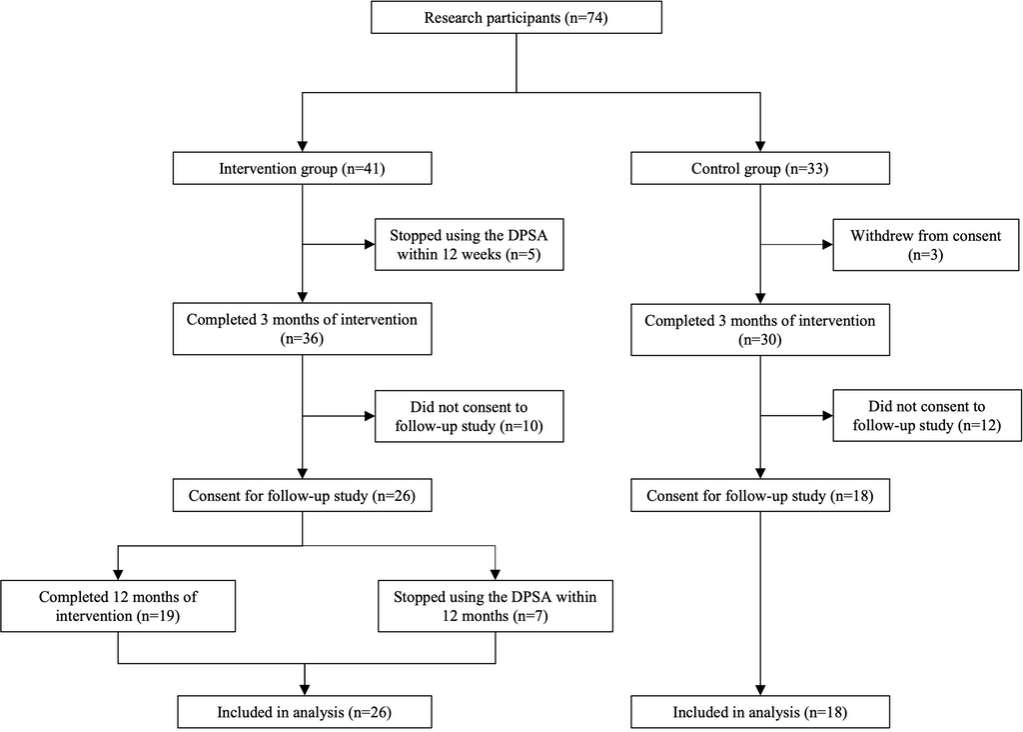
Flow diagram of participant enrollment and follow-up. DPSA: digital peer-supported app.

This study used Minchalle, a commercially available DPSA [51]. This mobile app was developed in June 2015 by A10 Lab Inc, with an initial release in November 2015. Figure 2 shows a sample app screen. The DPSA creates a group chat for up to 5 people with a common goal, and participants anonymously interact with each other in the group. The common goal of the intervention group was to increase their physical activity by walking and exercising. Once a day, participants posted their step counts, photographs, and comments in a group chat box. The main functions of the DPSA used in this study were as follows: (1) posting photographs, step counts, and comments about the day, (2) reaction buttons from group members (Figure 2), (3) setting step count goals on a group basis, and (4) providing feedback on the group’s total daily step count. Step counts were measured using a smartphone, and the DPSA reported the number of steps taken on that day at the time of posting. The participants were asked to carry smartphones throughout the day while they were awake. Participants had the option to post comments or photographs multiple times a day and engage with other members. The mobile app was provided to the participants free of charge. Details of the DPSA’s functionality are summarized in Multimedia Appendix 1.

### Characteristics of Research Participants

In addition to general characteristics, such as age and sex, the survey enquired about smartphone ownership, frequency of app use (except DPSA), and exercise habits. Body weight (kg) was measured using a digital scale, and height (m) was measured with a stadiometer after removing shoes. BMI was calculated as body weight divided by the square of height. Exercise habits were considered as “those who exercised at least twice a week for 30 minutes or longer each time for at least 1 year” [52].

The frequency of neighborhood interaction was assessed by asking the participants how many times they interact with people in the neighborhood within 1 week. Group exercise participation was defined as study participants who participate in a group of 3 or more people who meet voluntarily to exercise.

### DPSA Feasibility

DPSA feasibility was assessed based on retention and adherence rates during the year of program implementation. The retention rate indicates how many of the participants continued to use the DPSA for 12 months. The adherence rate indicates how often participants used the DPSA during the intervention period. The DPSA used in this study excludes a person from a group if they have not posted a set of step counts, photographs, or comments for 15 consecutive days. Dropouts were defined as those excluded from the group during the 12 months of DPSA use by researchers. Retention rates were calculated using a population of 26 participants in the intervention group and a population of all 41 participants who decided not to participate in the follow-up study. The retention rate was considered good if it was ≥70% (≥29 retention out of 41) based on previous studies by Farrance et al [53] and Picorelli et al [54]. The adherence rate was calculated by dividing the number of sets of step counts, photographs, and comments posted during the intervention period by the duration of the intervention. Adherence was calculated as the percentage of both participants, including dropouts and not including dropouts. Considering that the adherence rate for participants in the 3-month program was 87.7% [30], an adherence rate of ≥80% was considered good. The adherence rates were also calculated by group (7 groups: A-G). The number of all chat posts per person by group was calculated to assess the degree to which the group was used. The observed negative physical conditions during the intervention were ascertained by interviewing participants 12 months later. The app developers and the municipality were available to support the participants for any privacy breaches and technical issues.

### Outcome Measure

To assess physical activity, participants were asked to wear a triaxial accelerometer [55] (Active Style Pro HJA-750C activity meter; Omron Healthcare) at the waist level for 7 consecutive days for a total of 5 times every 3 months starting before the intervention. This accelerometer provides a relatively accurate measure of physical activity in healthy older adults [56]. Participants were instructed not to remove the device unless required for certain tasks, such as changing clothes and bathing. At the end of the measurement, all the data collected were transferred from the accelerometer to a personal computer. Following the suggested method [57] for estimating physical activity, an individual was required to record ≥10 hours of activity per day for 3 days to be included in the subsequent analyses. The data were collected in 60-second epochs for data analysis and used to estimate the intensity of the activity (METs). Outcome measurements of physical activity included the mean daily step count and time spent in sedentary behavior (SB: ≤1.5 METs), light-intensity physical activity (LPA: 1.6‐2.9 METs), and MVPA (≥3 METs) per day. The number of steps reported to the group chat in the DPSA was measured by the smartphone but was not used as an outcome.

Physical function was assessed using grip strength and the 30-second chair stand test (CS-30). The grip strength was measured using a digital dynamometer (Grip D; TKK 5401; Takei Scientific Instruments). This digital dynamometer was reliable and was validated relative to the Jamar dynamometer, which is the most frequently cited instrument for assessing grip strength in adults aged older than 60 years [58]. Measurements were taken in the standing position with the elbow joint in extension and the wrist joint in midextension. The left and right hands were measured once, and the highest value was used for data analysis. For the CS-30 test [59], seated participants were instructed to stand up from the chair with their arms crossed at the chest level as many times as possible in 30 seconds. The CS-30 has been reported to be quite reliable and valid as an indicator of lower-limb function in older adults [59].

Self-efficacy for exercise consisted of 4 questions on self-confidence in exercising under the following conditions [60]: physical fatigue, mental stress, lack of time, and bad weather. In response to the question, “Do you have the confidence to exercise regularly under the following conditions?” participants were asked to select 1 of 5 answers ranging from “No, I don’t have any confidence at all (1 point)” to “Yes, I am quite confident (5 points).” The total score ranged from 4 to 20.

### Statistical Analysis

This study used intention-to-treat analysis. Participant characteristics between groups were compared using independent sample *t*, chi-square, and Mann-Whitney *U* tests. Fixed-effects models were used because of the intensive repeated-measures design [61]. The advantage of this method is that it can handle nested observations, unbalanced numbers of observations, and missing values [62]. Although it would have been desirable to use a model that included random effects in this study, sample size limitations impeded the convergence of the mixed-effects model, and thus, we applied a model with fixed effects only. Yet, the fixed-effects model is still capable of capturing changes in the repeated measures in the outcomes. On a related note, linear mixed-effects models can be used with small sample sizes [63,64]. Between-group differences (intervention vs control) were analyzed using fixed-effects models adjusted for baseline age, sex, and app usage frequency (at baseline). The interaction between the groups and the time of the intervention was then analyzed.

Subsequently, the effects of the intervention for each group were analyzed using linear mixed-effects models, and significant differences compared with preintervention were evaluated using the Bonferroni method (accelerometer data were adjusted for wear time). Dependent variables, such as daily step count, SB, LPA, MVPA, grip strength, CS-30, and self-efficacy for exercise, were analyzed in separate models. Although the daily step count distribution did not precisely follow the normal distribution, the consistent results were obtained when applying a square root transformation, and, therefore, the results without the square root transformation are presented for interpretation. Data were analyzed using IBM SPSS Statistics 29 (IBM). The statistical significance level was set to 5%.

## Results

### Participants

The follow-up study included 44 of 66 participants from the 3-month intervention study. Of these, 26 were in the intervention group and 18 in the control group (Figure 3). The intervention group consisted of 7 groups of 3-4 people.

### Participant Characteristics

Table 1 shows the baseline participant characteristics. The mean age (SD) of the participants in the intervention group was 75.1 (5.1) years, with 13/26 men (50%). In the control group, the mean age was 77.4 (SD 5.3) years, with 6/18 men (33%). Although the participants were relatively older (average age of 76.0 years), both groups consisted of active older adults who had regular exercise habits, engaged in active interactions with their neighbors, and had no health problems that would interfere with study participation. No statistically significant differences were observed in the baseline demographic characteristics between the intervention and control groups. Although statistically significant differences were not observed arguably owing to the small sample sizes, the intervention group had larger proportions of smartphone ownership and app use frequency.

**Table 1.** Participant characteristics.

Characteristics	Total sample (n=44)	Intervention group (n=26)	Control group (n=18)	*P* value
Age (years), mean (SD**)**	76.0 (5.2)	75.1 (5.1)	77.4 (5.3)	.15^a^
Sex, n (%)				.27^b^
Male	19 (43)	13 (50)	6 (33)	
Female	25 (57)	13 (50)	12 (66)	
BMI (kg/m^2^), mean (SD)	22.9 (3.0)	23.3 (3.0)	22.2 (3.0)	.27^a^
Living alone, n (%)	11 (26)	8 (31)	3 (17)	.24^b^
Self-rated health, n (%)				.33^b^
Excellent, good, or normal	39 (89)	24 (92)	15 (83)	
Fair or poor	5 (11)	2 (8)	3 (17)	
Perceived household economic status, n (%)				.36^b^
Excellent, good, or normal	41 (93)	25 (96)	16 (89)	
Fair or poor	3 (7)	1 (3)	2 (7)	
Life satisfaction, n (%)				.52^b^
Excellent or good, or normal	38 (86)	22 (85)	16 (90)	
Fair or poor	7 (14)	4 (15)	3 (10)	
Working, n (%)	12 (28)	7 (27)	5 (28)	.61^b^
Smartphone owner, n (%)	41 (93)	26 (100.0)	15 (83)	.06^b^
Frequency of app use, n (%)				.09^b^
Usually or sometimes	34 (80)	24 (92)	11 (61)	
Rarely or never	9 (21)	2 (8)	7 (39)	
Exercise habits^c^, n (%)	24 (55)	16 (62)	8 (44)	.26^b^
Frequency of neighborhood interaction, n (%)				.30^b^
≥3 times per week	20 (45)	13 (50)	7 (39)	
≤2 times per week	24 (55)	13 (50)	11 (61)	
Participation in group exercise, n (%)	19 (43)	10 (38)	9 (50)	.45^b^
History falls in the past year, n (%)	6 (14)	3 (12)	3 (17)	.48^b^
Effect of COVID-19 on decreased physical activity, n (%)				.62^b^
Greatly/slightly	31 (70)	19 (73)	12 (67)	
Not much/unchanged	13 (30)	7 (27)	6 (33)	
Self-reported decrease in walking speed, n (%)	31 (70)	19 (73)	12 (67)	.65^b^
Triaxial accelerometer				
Steps per day, median (IQR)	6849 (4187‐8688)	7082 (4434‐9866)	5276 (4062‐7143)	.15^d^
LPA^e^ (minutes per day), mean (SD)	330.5 (89.5)	303 (72.1)	369.1 (98.8)	.03^a^
MVPA^f^ (minutes per day), mean (SD)	51.4 (27.9)	57.7 (25.3)	42.7 (29.7)	.047^a^
SB^g^ (minutes per day), mean (SD)	540.0 (113.4)	538.7 (85.8)	541.8 (146.2)	.82^a^
Triaxial accelerometer wearing time (minutes per day), mean (SD)	921.9 (115.6)	899.2 (66.6)	953.6 (157.8)	.18^a^
Physical function, mean (SD)				
Grip strength (kg)	26.4 (8.3)	28.1 (8.7)	24.2 (7.3)	.13^a^
CS-30^h^	20.1 (6.8)	20.4 (7.6)	19.6 (5.6)	.81^a^
Self-efficacy for exercise, mean (SD**)**	13.4 (3.4)	13.6 (3.2)	13.1 (3.2)	.47^a^

a Analysis was conducted using the independent samples *t* test (2-tailed).

b Analysis was conducted using the chi-squared test.

c Exercise habit was defined as exercising at least twice a week for ≥30 minutes each time for ≥1 year.

d Analysis was conducted using the Mann-Whitney *U* tests.

e LPA: light-intensity physical activity.

f MVPA: moderate to vigorous intensity physical activity.

g SB: sedentary behavior.

h CS-30: 30-second chair stand test.

### Feasibility: Retention Rate, Number of Posts, and Negative Impact

The retention rate among the 26 participants in the intervention group was 96% (25/26) in the 6th month, 92% (24/26) in the 9th month, and 73% (19/26) in the 12th month. The retention rate, based on 41 participants, all of whom did not participate in the follow-up survey, was 61% (25/41) in the 6th month, 59% (24/41) in the 9th month, and 46% (19/41) in the 12th month; thus, this retention rate was <70% at the beginning of the follow-up study. The reasons for dropping out of DPSA were “contracted COVID-19 and stopped submitting” (n=1), “unknown cause” (n=2), and “after discussion in a group chat, everyone stopped using DPSA” (n=4).

The adherence rate and number of total posts per day among members of the intervention group are summarized in Table 2. The adherence for the DPSA was 85.9%. The total number of chats per person averaged 2.55 (SD 1.28) per day. Excluding dropouts, the adherence rate was 92.3%, with a total of 2.88 (SD 1.24) posts per day per person. Adherence rates were good among participants in the follow-up study. One group had all members drop out; all members of the group were male. Three cases of mild physical discomfort that did not interfere with daily life were reported, with 2 participants reporting knee pain and 1 reporting foot pain.

**Table 2.** Digital peer-supported app adherence rate and number of total posts per day among members of the intervention group.

	All participants (n=26)		Excluding dropout (n=19)	
Group	Adherence rate, n (%)	Total posts/person /day, mean (SD)	Adherence rate, n (%)	Total posts/person /day, mean (SD)
All	26 (85.9)	2.55 (1.28)	19 (92.3)	2.88 (1.24)
A	4 (86.3)	1.40 (0.33)	4 (86.3)	1.40 (0.33)
B	4 (95.4)	2.61 (0.77)	3 (99.1)	2.63 (0.94)
C	4 (75.0)	1.17 (0.41)	All dropouts	All dropouts
D	4 (98.6)	2.24 (0.58)	4 (98.6)	2.24 (0.58)
E	3 (84.7)	4.17 (0.22)	3 (84.7)	4.17 (0.22)
F	4 (85.4)	3.28 (0.63)	4 (85.4)	3.28 (0.63)
G	3 (75.9)	3.66 (2.10)	2 (99.6)	4.74 (1.38)

No privacy breaches were associated with app usage. There were 2 inquiries from participants, including account transfer after a smartphone model change (about 30 minutes) and uninstallation of the DPSA (about 20 minutes).

### Changes in Physical Activity and Function and Self-Efficacy for Exercise

Table 3 shows the analysis results. In the group comparison of the linear mixed-effects model analyses of physical activity and function and self-efficacy for exercise, no differences were observed. However, a significant change was observed in step count over time only in the intervention group (*P*=.048), wherein we observed an increase of 1736 (β=1736, 95% CI 232-3241) steps per day compared with baseline. LPA and SB showed differences in the control group, but no significant difference was noted at any time point compared with baseline. Regarding the CS-30, there was a significant within-group difference in the increase in CS-30 scores for the intervention (*P*<.001) and control (*P*=.03) groups over the 12-month period. Additionally, the change in CS-30 scores between the baseline and 12-month assessments was 6.5 (β=6.5, 95% CI 3.8-9.1) times in the intervention group (*P*<.001) and 3.8 (β=3.8, 95% CI 0.6-7.1) times in the control group (*P*=.02). Regarding the self-efficacy for exercise, a significant change over time was observed only in the intervention group (*P*=.03), wherein an increase of 1.6 (β=1.6, 95% CI 0.2-3.1) points was observed after 12 months compared with baseline (*P*=.03).

**Table 3.** Included outcome measures at baseline and 3, 6, and 12 months with within-group and between-group comparisons (26 participants in the intervention group and 18 participants in the control group).

Outcome measures	Intervention			Control			Group × time^a^
	Comparison with baseline		Within-group changes	Comparison with baseline		Within-group changes	
	β (95% CI)	*P* value	*P* value	β (95% CI)	*P* value	*P* value	*P* value
Steps per day^b^			.048			.08	.25
Baseline	Reference						
3 months	960 (−505 to 2425)	.39		150 (−765 to 1065)	.99		
6 months	1213 (−231 to 2657)	.14		274 (−633 to 1181)	.99		
9 months	581 (−910 to 2072)	.99		−653 (−1566 to 260)	.28		
12 months	1736 (232 to 3241)	.02		147 (−803 to 1096)	.99		
LPA^b^^,^^c^ (minutes per day)			.18			.044	.71
Baseline	Reference						
3 months	−6 (−35 to 23)	.99		−21 (−49 to 7)	.23		
6 months	16 (−13 to 44)	.67		3 (−24 to 31)	.99		
9 months	5 (−25 to 35)	.99		−16 (−44 to 12)	.57		
12 months	18 (−12 to 48)	.048		7 (−22 to 36)	.99		
MVPA^b^^,^^d^ (minutes per day)			.07			.96	.28
Baseline	Reference						
3 months	14 (1 to 26)	.02		1 (−7 to 10)	.99		
6 months	8 (−4 to 21)	.32		0 (−8 to 8)	.99		
9 months	5 (−7 to 18)	.99		−1 (−9 to 7)	.99		
12 months	10 (−3 to 22)	.23		0 (−9 to 8)	.99		
SB^b^^,^^e^ (minutes per day)			.16			.049	.62
Baseline	Reference						
3 months	−8 (−40 to 24)	.99		20 (−8 to 48)	.30		
6 months	−24 (−56 to 7)	.22		−4 (−31 to 24)	.99		
9 months	−10 (−43 to 22)	.99		17 (−11 to 45)	.47		
12 months	−28 (−61 to 5)	.13		−6 (−36 to 23)	.99		
Triaxial accelerometer wearing time (minutes per day)			.51			.20	.27
Baseline	Reference						
3 months	9 (−38 to 56)	.99		−26 (−70 to 19)	.59		
6 months	−8 (−54 to 39)	.99		−14 (−58 to 31)	.99		
9 months	−19 (−67 to 28)	.99		−23 (−68 to 21)	.73		
12 months	9 (−39 to 58)	.99		−42 (−87 to 4)	.09		
Grip strength (kg)			.09			.15	.12
Baseline	Reference						
3 months	−0.8 (−2.1 to 0.5)	.31		−0.9 (−2.5 to 0.7)	.35		
12 months	−1.1 (−2.3 to 0.1)	.07		−6 (−36 to 23)	.95		
CS-30 (times)^b^^,^^f^			<.001			.03	.41
Baseline	Reference						
3 months	1.4 (−1.1 to 4.0)	.40		0.6 (−2.1 to 3.3)	.99		
12 months	6.5 (3.8 to 9.1)	<.001		3.8 (0.6 to 7.1)	.02		
Self-efficacy for exercise (points)			.03			.54	.53
Baseline	Reference						
3 months	1.1 (−0.2 to 2.5)	.12		0.3 (−0.8 to 1.5)	.99		
12 months	1.6 (0.2 to 3.1)	.02		0.7 (−0.8 to 2.1)	.58		

a Analyses were adjusted for age, sex, and frequency of app use (baseline).

b Triaxial accelerometer data were adjusted for wear time.

c LPA: light-intensity physical activity.

d MVPA: moderate to vigorous intensity physical activity.

e SB: sedentary behavior.

f CS-30: 30-second chair stand test.

## Discussion

### Principal Results

This study aimed to confirm the feasibility of a 12-month intervention using the DPSA to improve physical activity among older adults and to obtain preliminary estimates of its effects on physical activity, physical function, and self-efficacy for exercise. The retention rate in the intervention group (n=26) over the 12-month period was 73% (19/26). Considering the 41 participants who did not express interest in the follow-up surveys as the denominators, the retention rate was 46% (19/41). The adherence rate was 87.7%. This study obtained preliminary estimates of the effects of DPSA use on physical activity, physical function, and self-efficacy for exercise.

### Comparison With Previous Studies

This is a rare study that examined the 12-month long-term feasibility and changes of an app intervention aimed at promoting physical activity in older adults. The 26 participants in the intervention group who used the DPSA had a 12-month retention rate of 73%. Including participants who did not indicate a desire for a follow-up survey, the 12-month retention rate was 46%. A previous study that used a smartphone app and smart band for weight loss, physical activity, and caloric intake in an overweight and obese population aged between 20 and 65 years for 12 months reported a 12-month retention rate of 68.4% (227/332) [65]. Moreover, a previous study of adults aged 30‐60 years on physical activity and weight loss in Japan using a smartphone app focused on steps reported a 12-month retention rate of 95% (52/55) [66]. Compared with that reported by these previous studies, the 12-month retention rate was lower. The low retention rate might have been because the study included older adults who were less familiar with the app than younger adults [67], and daily posting may have been stressful for participants with the limited app experience. Only about half of the older adults were capable of long-term retention in the DPSA. One of the 7 groups had all its members drop out; this group was unique in that all members were males. Groups comprising a mix of male and female members may last longer. Group chat members with fewer posts, indicating lower engagement, tended to drop out. The following strategies can be adopted to prevent dropouts: providing opportunities for interactions among group members, encouraging people to make supportive posts to each other, and providing canned messages, such as greeting and appreciation messages, to allow group chat members communicate with each other through simple operations. Only 3 negative physical effects were reported; however, they were all minor and did not cause privacy issues.

In this study, significant changes in the number of steps taken and the self-efficacy for exercise score (Table 3) were observed within the intervention group, but there was no significant difference between groups. Self-efficacy is an important aspect of social cognitive theory [23]. As hypothesized, peer support based on social cognitive theory improves self-efficacy for exercise, resulting in increased steps. In the intervention group, an increase of more than 1000 average daily steps was observed. Increasing the number of steps taken daily by ≥1000 reduces the risk of various diseases and mortality [68,69] . A systematic review of 17 prospective studies by Hall et al [68] showed that each 1000-step increase in the daily step count decreases the risk of death and heart disease, with a 6%‐36% decrease in all-cause mortality risk and a 5%‐21% decrease in heart disease risk. Furthermore, an increase of 1000 steps per day decreases a woman’s risk of diabetes by 6% and an increase of 2000 steps per day decreases the risk of diabetes by 12% [69]. Although there was an increase in MVPA in the 3-month intervention, there were no significant differences within groups in this study. However, MVPA increased by 10 (95% CI −3 to 22) minutes per day at 12 months compared with baseline. This result may be an effect of the small sample size. Peer support can build trust and provide social support through interpersonal communication [70]. In peer-based intervention strategies aimed at promoting physical activity among older adults, social support is considered a key factor in facilitating behavior change [71]. In this study, social support through peer support may have influenced physical activity levels. However, the evaluation of social support provided by the DPSA was lacking and should be addressed in future research.

In this study, CS-30 scores changed from baseline to 12 months for both intervention and control groups (Table 3), but no significant differences were observed between groups. In the intervention group, long-term continuation of the DPSA may have increased self-efficacy for exercise and the number of steps taken, leading to improved lower limb function. The DPSA may be effective as a means to improve lower limb function. This is a meaningful result because improving lower limb function may lead to the prevention of falls [72,73], sarcopenia [9,74,75], frailty [74,76], and dementia [34,72,77], and may also lead to reductions in health care costs associated with these conditions [78]. The control group also improved lower extremity function with an increase in CS-30 scores. Older adults in the control group did not use the DPSA. They attended exercise instruction and continued regular physical activity monitoring with an accelerometer. The improvement in lower limb function may have been due to voluntary physical activity or strength training that could not be adequately measured with an activity meter. The DPSA is not for everyone, as it requires possession of a smartphone and an understanding of its usage. It may be important to select a menu of interventions that is tailored to the characteristics of the participants.

### Limitations

This study has the following 4 limitations. First, the study design was less capable of demonstrating the effects of the DPSA, compared with an RCT. Participants were nonrandomly assigned to the intervention and control groups and free to choose the group in which they would participate. Older adults who did not own a smartphone were unable to participate in the intervention group, and those unfamiliar with the app’s operation were less likely to join. Given that this was a non-RCT, fully accounting for possible confounding bias was challenging, making it difficult to accurately estimate the intervention’s effect by comparing the 2 groups. Additionally, follow-up participation was voluntary. The use of the DPSA is applicable to only eligible older Fujisawa City residents who own smartphones and are interested in mobile apps.

Second, the sample size was small. The follow-up participation rate was 63% (26/41) in the intervention group and 55% (18/33) in the control group. This low participation rate reduces the study’s validity and may have impacted the feasibility and estimates of the effects on physical activity, physical function, and self-efficacy for exercise. The small sample size might have resulted in insufficient statistical power to detect differences between groups, and the model parameter estimation might have been unstable. Furthermore, the small sample size did not allow the convergence of the mixed-effects model. The older adults in this study took more steps per day and were originally sufficiently physically active compared with the general healthy older adult population [79]. The mean baseline score for adults in Japan aged 60 years or older for CS-30 score was 17.3 times [80]. At baseline, the intervention group averaged 20.4 times and the control group averaged 19.6 times. The study participants originally had the adequate level of lower extremity function. Future studies may benefit an aging society by targeting many older adults who are less physically active and have poor lower extremity function.

Third, given that this study included only those who participated in the follow-up, survival bias may have been present. In the intervention group, participants who did not complete the follow-up study were older and engaged in less physical activity at baseline than those who did. Therefore, the feasibility findings and estimates of changes in physical activity, physical function, and self-efficacy for exercise may be overestimated.

Fourth, the generalizability of this study is limited owing to potential selection bias. Participants in the intervention group were not only motivated to increase physical activity but also familiar with using the app. The DPSA was not adaptable to all participants, as it required a certain level of information technology literacy and cognitive function. Social, cultural, and economic factors (eg, older age, privacy concerns, and low income) may influence preference and feasibility with smartphone apps [81-83]. Therefore, the use of DPSA may not be widely accepted.

### Conclusions

This study assessed the 12-month feasibility of using the DPSA and measured preliminary estimates of its effects on physical activity, physical function, and self-efficacy for exercise. The 12-month retention rate for participants in the DPSA intervention group was 73% (19/26), and that for 41 participants including those who decided not to participate in the follow-up study was 46% (19/41). The DPSA adherence rate was 85.9%. Only a limited number of older adults had long-term access to the DPSA. Preliminary estimates suggest that DPSA use may improve step count, lower extremity function, and self-efficacy for exercise. However, various biases were introduced, preventing the demonstration of clear intervention effects. There is a need to identify ways in which more older adults can use DPSAs for extended periods of time; RCTs should be conducted to ascertain the long-term effects of DPSAs on physical activity and function in older adults.

## Supplementary material

10.2196/66610 Multimedia Appendix 1 Details of the digital peer-supported app’s functionality.

## Abbreviations

BMI: body mass index
CS-30: 30-second chair stand test
DPSA: digital peer-supported app
IQR: interquartile range
LPA: light-intensity physical activity
MET: metabolic equivalent
MVPA: moderate to vigorous intensity physical activity
RCT: randomized controlled trial
SB: sedentary behavior

## References

[1] Zhang D (2024). Perceived neighborhood conditions, psychosocial factors, and sleep problems among urban and rural older adults in China. J Aging Health.

[2] Lutz W, Sanderson W, Scherbov S (1997). Doubling of world population unlikely. Nature New Biol.

[3] Lang PO, Govind S, Aspinall R (2013). Reversing T cell immunosenescence: why, who, and how. Age (Dordr).

[4] Bouaziz W, Vogel T, Schmitt E, Kaltenbach G, Geny B, Lang PO (2017). Health benefits of aerobic training programs in adults aged 70 and over: a systematic review. Arch Gerontol Geriatr.

[5] (2023). White paper on the aging society. Cabinet Office Japan.

[6] (2022). Dementia. World Health Organization.

[7] Katzmarzyk PT, Friedenreich C, Shiroma EJ, Lee IM (2022). Physical inactivity and non-communicable disease burden in low-income, middle-income and high-income countries. Br J Sports Med.

[8] Manning KM, Hall KS, Sloane R (2024). Longitudinal analysis of physical function in older adults: the effects of physical inactivity and exercise training. Aging Cell.

[9] Wang H, Huang WY, Zhao Y (2022). Efficacy of exercise on muscle function and physical performance in older adults with sarcopenia: an updated systematic review and meta-analysis. Int J Environ Res Public Health.

[10] Lee IM, Shiroma EJ, Lobelo F (2012). Effect of physical inactivity on major non-communicable diseases worldwide: an analysis of burden of disease and life expectancy. Lancet.

[11] (2024). Promotion of physical activity and exercise 2023. Ministry of Health, Labour and Welfare.

[12] Dipietro L, Campbell WW, Buchner DM (2019). Physical activity, injurious falls, and physical function in aging: an umbrella review. Med Sci Sports Exerc.

[13] Katz S (1983). Assessing self-maintenance: activities of daily living, mobility, and instrumental activities of daily living. J Am Geriatr Soc.

[14] Rejeski WJ, Ip EH, Marsh AP, Miller ME, Farmer DF (2008). Measuring disability in older adults: the International Classification System of Functioning, Disability and Health (ICF) framework. Geriatr Gerontol Int.

[15] Fukuoka Y, Haskell W, Lin F, Vittinghoff E (2019). Short- and long-term effects of a mobile phone app in conjunction with brief in-person counseling on physical activity among physically inactive women: the mPED randomized clinical trial. JAMA Netw Open.

[16] Laranjo L, Ding D, Heleno B (2021). Do smartphone applications and activity trackers increase physical activity in adults? Systematic review, meta-analysis and metaregression. Br J Sports Med.

[17] Moss RJ, Süle A, Kohl S (2019). eHealth and mHealth. Eur J Hosp Pharm.

[18] Elavsky S, Knapova L, Klocek A, Smahel D (2019). Mobile health interventions for physical activity, sedentary behavior, and sleep in adults aged 50 years and older: a systematic literature review. J Aging Phys Act.

[19] Kwan RYC, Salihu D, Lee PH (2020). The effect of e-health interventions promoting physical activity in older people: a systematic review and meta-analysis. Eur Rev Aging Phys Act.

[20] D’Amore C, Reid JC, Chan M (2022). Interventions including smart technology compared with face-to-face physical activity interventions in older adults: systematic review and meta-analysis. J Med Internet Res.

[21] Duan Y, Shang B, Liang W, Du G, Yang M, Rhodes RE (2021). Effects of eHealth-based multiple health behavior change interventions on physical activity, healthy diet, and weight in people with noncommunicable diseases: systematic review and meta-analysis. J Med Internet Res.

[22] Morey MC, Snyder DC, Sloane R (2009). Effects of home-based diet and exercise on functional outcomes among older, overweight long-term cancer survivors: RENEW: a randomized controlled trial. JAMA.

[23] Bandura A (1986). Social Foundations of Thought and Action: A Social Cognitive Theory.

[24] McCullagh P, Weiss MR, Ross D (1989). Modeling considerations in motor skill acquisition and performance: an integrated approach. Exerc Sport Sci Rev.

[25] Posavac EJ, Kattapong KR, Dew DE (1999). Peer-based interventions to influence health-related behaviors and attitudes: a meta-analysis. Psychol Rep.

[26] Ashford D, Bennett SJ, Davids K (2006). Observational modeling effects for movement dynamics and movement outcome measures across differing task constraints: a meta-analysis. J Mot Behav.

[27] McAuley E, Blissmer B (2000). Self-efficacy determinants and consequences of physical activity. Exerc Sport Sci Rev.

[28] Ginis KAM, Nigg CR, Smith AL (2013). Peer-delivered physical activity interventions: an overlooked opportunity for physical activity promotion. Transl Behav Med.

[29] Webel AR, Okonsky J, Trompeta J, Holzemer WL (2010). A systematic review of the effectiveness of peer-based interventions on health-related behaviors in adults. Am J Public Health.

[30] Tabira K, Oguma Y, Yoshihara S (2024). Digital peer-supported app intervention to promote physical activity among community-dwelling older adults: nonrandomized controlled trial. JMIR Aging.

[31] van der Meulen K, Granizo L, Del Barrio C (2021). Emotional peer support interventions for students with SEND: a systematic review. Front Psychol.

[32] Burton E, Farrier K, Hill KD, Codde J, Airey P, Hill AM (2018). Effectiveness of peers in delivering programs or motivating older people to increase their participation in physical activity: systematic review and meta-analysis. J Sports Sci.

[33] Iliffe S, Kendrick D, Morris R (2010). Multi-centre cluster randomised trial comparing a community group exercise programme with home based exercise with usual care for people aged 65 and over in primary care: protocol of the ProAct 65+ trial. Trials.

[34] McGarrigle L, Todd C (2020). Promotion of physical activity in older people using mHealth and eHealth technologies: rapid review of reviews. J Med Internet Res.

[35] Royse LA, Baker BS, Warne-Griggs MD (2023). “It’s not time for us to sit down yet”: how group exercise programs can motivate physical activity and overcome barriers in inactive older adults. Int J Qual Stud Health Well-being.

[36] Reiner M, Niermann C, Jekauc D, Woll A (2013). Long-term health benefits of physical activity—a systematic review of longitudinal studies. BMC Public Health.

[37] Jonkman NH, van Schooten KS, Maier AB, Pijnappels M (2018). eHealth interventions to promote objectively measured physical activity in community-dwelling older people. Maturitas.

[38] Yerrakalva D, Yerrakalva D, Hajna S, Griffin S (2019). Effects of mobile health app interventions on sedentary time, physical activity, and fitness in older adults: systematic review and meta-analysis. J Med Internet Res.

[39] Mönninghoff A, Kramer JN, Hess AJ (2021). Long-term effectiveness of mHealth physical activity interventions: systematic review and meta-analysis of randomized controlled trials. J Med Internet Res.

[40] Stecher C, Pfisterer B, Harden SM (2023). Assessing the pragmatic nature of mobile health interventions promoting physical activity: systematic review and meta-analysis. JMIR Mhealth Uhealth.

[41] Nyman SR, Victor CR (2012). Older people’s participation in and engagement with falls prevention interventions in community settings: an augment to the Cochrane systematic review. Age Ageing.

[42] Olanrewaju O, Kelly S, Cowan A, Brayne C, Lafortune L (2016). Physical activity in community dwelling older people: a systematic review of reviews of interventions and context. PLoS One.

[43] (2023). Fujisawa City future population estimates. Fujisawa City.

[44] Landry R, Traore N, Godin B (1996). An econometric analysis of the effect of collaboration on academic research productivity. High Educ.

[45] Dooley L, Kirk D (2007). University‐industry collaboration. Eur J Innov Manage.

[46] Thomas S, Reading J, Shephard RJ (1992). Revision of the Physical Activity Readiness Questionnaire (PAR-Q). Can J Sport Sci.

[47] Bredin SSD, Gledhill N, Jamnik VK, Warburton DER (2013). PAR-Q+ and ePARmed-X+: new risk stratification and physical activity clearance strategy for physicians and patients alike. Can Fam Physician.

[48] (2013). Active guide: Japanese official physical activity guidelines for health promotion brochure in English. Ministry of Health, Labour and Welfare.

[49] (2014). Fujisawa +10 exercise. Fujisawa City Health and Medical Center.

[50] Osawa Y, Saito Y, Tsunekawa N, Manabe T, Oguma Y (2015). Exercise workload of the “Fujisawa +10 exercise” program in older women. J Exer Physiol Online.

[51] (2023). Minchalle Web. A10Lab. Inc.

[52] (2017). Ministry of Health, Labour and Welfare. National health and nutrition survey.

[53] Farrance C, Tsofliou F, Clark C (2016). Adherence to community based group exercise interventions for older people: a mixed-methods systematic review. Prev Med.

[54] Picorelli AMA, Pereira LSM, Pereira DS, Felício D, Sherrington C (2014). Adherence to exercise programs for older people is influenced by program characteristics and personal factors: a systematic review. J Physiother.

[55] Ohkawara K, Oshima Y, Hikihara Y, Ishikawa-Takata K, Tabata I, Tanaka S (2011). Real-time estimation of daily physical activity intensity by a triaxial accelerometer and a gravity-removal classification algorithm. Br J Nutr.

[56] Park J, Ishikawa-Takata K, Tanaka S, Bessyo K, Tanaka S, Kimura T (2017). Accuracy of estimating step counts and intensity using accelerometers in older people with or without assistive devices. J Aging Phys Act.

[57] Jefferis BJ, Sartini C, Ash S (2015). Trajectories of objectively measured physical activity in free-living older men. Med Sci Sports Exerc.

[58] Savas S, Kilavuz A, Kayhan Koçak FÖ, Cavdar S (2023). Comparison of grip strength measurements by widely used three dynamometers in outpatients aged 60 years and over. J Clin Med.

[59] Jones CJ, Rikli RE, Beam WC (1999). A 30-s chair-stand test as a measure of lower body strength in community-residing older adults. Res Q Exerc Sport.

[60] Oka K (2003). Stages of change for exercise behavior and self-efficacy for exercise among middle-aged adults. Nihon Koshu Eisei Zasshi.

[61] Li Y, Baron J, Li Y, Baron J (2012). Behavioral Research Data Analysis With R.

[62] Raudenbush SW, Xiao-Feng L (2001). Effects of study duration, frequency of observation, and sample size on power in studies of group differences in polynomial change. Psychol Methods.

[63] Muth C, Bales KL, Hinde K, Maninger N, Mendoza SP, Ferrer E (2016). Alternative models for small samples in psychological research: applying linear mixed effects models and generalized estimating equations to repeated measures data. Educ Psychol Meas.

[64] Wiley RW, Rapp B (2019). Statistical analysis in Small-N Designs: using linear mixed-effects modeling for evaluating intervention effectiveness. Aphasiology.

[65] Lugones-Sanchez C, Recio-Rodriguez JI, Agudo-Conde C (2022). Long-term effectiveness of a smartphone app combined with a smart band on weight loss, physical activity, and caloric intake in a population with overweight and obesity (evident 3 study): randomized controlled trial. J Med Internet Res.

[66] Yoshimura E, Tajiri E, Michiwaki R, Matsumoto N, Hatamoto Y, Tanaka S (2022). Long-term effects of the use of a step count-specific smartphone app on physical activity and weight loss: randomized controlled clinical trial. JMIR Mhealth Uhealth.

[67] Mubarak F, Suomi R (2022). Elderly forgotten? Digital exclusion in the information age and the rising grey digital divide. Inquiry.

[68] Hall KS, Hyde ET, Bassett DR (2020). Systematic review of the prospective association of daily step counts with risk of mortality, cardiovascular disease, and dysglycemia. Int J Behav Nutr Phys Act.

[69] Garduno AC, LaCroix AZ, LaMonte MJ (2022). Associations of daily steps and step intensity with incident diabetes in a prospective cohort study of older women: the OPACH Study. Diabetes Care.

[70] Matz-Costa C, Howard EP, Castaneda-Sceppa C, Diaz-Valdes Iriarte A, Lachman ME (2019). Peer-based strategies to support physical activity interventions for older adults: a typology, conceptual framework, and practice guidelines. Gerontologist.

[71] Mead S, Hilton D, Curtis L (2001). Peer support: a theoretical perspective. Psychiatr Rehabil J.

[72] Ikegami S, Takahashi J, Uehara M (2019). Physical performance reflects cognitive function, fall risk, and quality of life in community-dwelling older people. Sci Rep.

[73] Sanchez M, Vidal JS, Bichon A (2023). Impact of a public open-access community-based physical activity and fall prevention program on physical performance in older adults. Eur J Public Health.

[74] Buchman AS, Leurgans SE, Wang T (2021). Motor function is the primary driver of the associations of sarcopenia and physical frailty with adverse health outcomes in community-dwelling older adults. PLoS One.

[75] Sawada S, Ozaki H, Natsume T (2021). The 30-s chair stand test can be a useful tool for screening sarcopenia in elderly Japanese participants. BMC Musculoskelet Disord.

[76] Pilotto A, Custodero C, Maggi S, Polidori MC, Veronese N, Ferrucci L (2020). A multidimensional approach to frailty in older people. Ageing Res Rev.

[77] Doi T, Tsutsumimoto K, Nakakubo S (2019). Physical performance predictors for incident dementia among Japanese community-dwelling older adults. Phys Ther.

[78] Arai H, Ouchi Y, Toba K (2015). Japan as the front-runner of super-aged societies: perspectives from medicine and medical care in Japan. Geriatr Gerontol Int.

[79] Tudor-Locke C, Craig CL, Aoyagi Y (2011). How many steps/day are enough? For older adults and special populations. Int J Behav Nutr Phys Act.

[80] Nakazono T, Kamide N, Ando M (2014). The reference values for the chair stand test in healthy Japanese older people: determination by meta-analysis. J Phys Ther Sci.

[81] Hoehle H, Zhang X, Venkatesh V (2015). An espoused cultural perspective to understand continued intention to use mobile applications: a four-country study of mobile social media application usability. Eur J Inf Syst.

[82] Gera R, Chadha P, Ahuja V (2020). Mobile app usage and adoption: a literature review. Int J Electron Bus.

[83] Wang C, Qi H (2021). Influencing factors of acceptance and use behavior of mobile health application users: systematic review. Healthcare (Basel).

