# Supplementary material for: A 12-Month Digital Peer-Supported App Intervention to Promote Physical Activity Among Community-Dwelling Older Adults: Follow-Up Study of a Nonrandomized Controlled Trial
Source: JMIR Aging. 2025 May 28;8:e66610. doi: 10.2196/66610 (PMC12136513; doi:10.2196/66610)
Supplement: Multimedia Appendix 1 [file aging-v8-e66610-s001.docx]

**"Minchalle" App**

1. **Posting to Chat has Four Functions**

- Challenge Photo
- Normal Photo
- Comment
- Stamp

1. **Exchange for Coins**
   - Function for Donation
2. **Function for Recording**
   - Records List
   - My Data
   - Calendar
3. **Assistant Robot**
4. **Posting to Chat has Four Functions**

**・Challenge Photo**

"Challenge photos" can be posted once a day and must be taken on the same day, with a limit of one submission per day. You will earn coins when your team members approve your challenge photos. To post a comment, follow these steps:

**・Normal Photo**

"Normal photos" can be posted as often as you like, and these can include images from the past. To post a comment, follow these steps:

**＊Difference between Challenge Photo and Normal Photo**

|  | Challenge Photo | Normal Photo |
| --- | --- | --- |
| Number of posts | Limited to once a day | Unlimited |
| Useable photos | Only photos taken on the same day | Past photos are acceptable |
| Display of the number of steps* | ◯ | ✖︎ |
| Approval button | ◯ | ✖︎ |
| Comment | ◯ | ✖︎ |

*Note： This applies only if you are part of a team that tracks your steps.

Note： A Enter steps; B Enter comments; C Steps and remarks after posting; D Approval button.

**・Comment**

Users can leave comments on the chat screen. Your comments will appear highlighted in red, whereas comments from other members will be in white. To post a comment, follow these steps:

**・Stamp**

Users can post stamps on the chat screen.

**2. Exchanged for Coins**

The app enables users to accumulate coins that can be redeemed for in-app wallpaper or stamps. Additionally, users have the option to utilize the app's donation function.

**・Donation function**

Coins can be donated to local governments and various organizations for their donation projects.

1. **Function for Recording**

The user's records can be traced back to the following three key points:

**・List of Past Records**

Track a record of the number of steps you have contributed to the past as well as the total number of steps taken by all participating teams.

**・My Data**

Users can evaluate their performance in achieving their set goals.

**・Calendar**

　　　Users can access a calendar to review their past challenge photo.

1. **Assistant Robot**

The team has access to an assistant robot that provides encouragement and feedback on their daily tasks. Team members can select and change their assistant robot as needed. However, the robot is not equipped to respond to user inquiries. These assistant robots are designed with distinct personalities, each tailored to enhance the team's experience. Here are the details of the team's robot's personality:

(A) Red assistant robots are designed to motivate and inspire users, developing good habits.

(B) Blue assistant robots focus on helping users create and maintain daily habits effectively.

(C) Green assistant robots support users in developing desired habits at their own pace.

(D) Yellow assistant robots aim to make the process of developing desirable habits enjoyable.

(E) Pink assistant robots are gentle and supportive, assisting users in creating desired habits.

(F) Black assistant robots have a more challenging approach and interfere with their activities.

1. **Team owner function**

　　Note that only the owner of the team has the authority to set or modify the following four points:

**・Team Name**

When forming a team of up to five members, the team can select a name. However, only the team owner has the authority to change the team’s name within the application.

**・Team Goals**

When forming a team with up to five members, the owner can set a team goal, such as achieving 10,000 steps per day. However, only the owner can modify the team's goal within the application.

**・Assistant Robot**

When creating a team of up to five members, the owner can select an assistant robot, such as a green assistant robot. However, only the owner has the authority to change the team's assistant robot within the application.

**・Automatic exit function**

To ensure active participation, each team should have the capability to automatically remove members who have not made a post within a specified time frame. Teams can select this time frame from the available options: "2 days," "4 days," "8 days," or "15 days."

**The actual operating procedure as follows:**
